# Associations between gait performance and pain intensity, psychosocial factors, executive functions as well as prefrontal cortex activity in chronic low back pain patients: A cross-sectional fNIRS study

**DOI:** 10.3389/fmed.2023.1147907

**Published:** 2023-05-05

**Authors:** Toan Nguyen, Martin Behrens, Kim-Charline Broscheid, Robert Bielitzki, Saskia Weber, Saskia Libnow, Victoria Malczewski, Lukas Baldauf, Xenia Milberger, Lena Jassmann, Anne Wustmann, Katharina Meiler, Steffen Drange, Jörg Franke, Lutz Schega

**Affiliations:** ^1^Department of Sport Science, Institute III, Otto von Guericke University Magdeburg, Magdeburg, Germany; ^2^Department of Orthopaedic Surgery, Klinikum Magdeburg gGmbH, Magdeburg, Germany

**Keywords:** spatio-temporal gait parameters, near-infrared spectroscopy, pain coping, PFC, single task and dual task walking

## Abstract

**Introduction:**

Activities of daily living, such as walking, are impaired in chronic low back pain (CLBP) patients compared to healthy individuals. Thereby, pain intensity, psychosocial factors, cognitive functioning and prefrontal cortex (PFC) activity during walking might be related to gait performance during single and dual task walking (STW, DTW). However, to the best of our knowledge, these associations have not yet been explored in a large sample of CLBP patients.

**Method:**

Gait kinematics (inertial measurement units) and PFC activity (functional near-infrared spectroscopy) during STW and DTW were measured in 108 CLBP patients (79 females, 29 males). Additionally, pain intensity, kinesiophobia, pain coping strategies, depression and executive functioning were quantified and correlation coefficients were calculated to determine the associations between parameters.

**Results:**

The gait parameters showed small correlations with acute pain intensity, pain coping strategies and depression. Stride length and velocity during STW and DTW were (slightly to moderately) positively correlated with executive function test performance. Specific small to moderate correlations were found between the gait parameters and dorsolateral PFC activity during STW and DTW.

**Conclusion:**

Patients with higher acute pain intensity and better coping skills demonstrated slower and less variable gait, which might reflect a pain minimization strategy. Psychosocial factors seem to play no or only a minor role, while good executive functions might be a prerequisite for a better gait performance in CLBP patients. The specific associations between gait parameters and PFC activity during walking indicate that the availability and utilization of brain resources are crucial for a good gait performance.

## 1. Introduction

Low back pain (LBP) is one of the most common medical problems worldwide (1). While it disappears in approximately 90% of the cases in the first few weeks, some people develop chronic low back pain (CLBP) episodes, which are defined as a pain duration of ≥3 months (2).

While CLBP is usually a consequence of anatomical or physiological anomalies (3), the psychosocial factors also seem to play a central role in the development of chronicity (2). Indeed, depression (4) and kinesiophobia (5, 6) are known to affect rehabilitation and, in some cases, worsen the CLBP symptoms. Further, these factors contribute to postural dysfunction, increased trunk stiffness, altered activation patterns of the abdominal and back extensor muscles (7), impaired motor control (8) and altered proprioception (9). These impairments can result in a decreased gait performance, which is crucial for activities of daily living (10).

In general, walking is a highly automatized motor activity that, depending on the demands, requires more or less additional attentional and cognitive resources (11). These demands further increase during the concurrent execution of walking and a cognitive task (motor-cognitive dual task), which often leads to a decline in motor and/or cognitive performance compared to the respective single task condition (dual task interference) (12). The spatio-temporal gait parameters, like stride length, velocity (13) and minimum toe clearance (14), recorded during single task and in particular during dual task conditions are of clinical relevance, given that a poor gait performance is related to the risk of falling (15). CLBP patients are especially affected by dual task interference (16), since acute and chronic pain have been shown to divert attention from other demands (17) and were associated with impairments in executive functions (EFs) (18, 19).

Additionally, it has been demonstrated that the prefrontal cortex (PFC) is not only involved in executive functioning, but also in pain processing (20) and prioritization during multiple attention-demanding tasks (11). The activity of the PFC during motor-cognitive dual tasks can be quantified using functional near-infrared spectroscopy (fNIRS), which is robust to motion artifacts compared to other portable techniques like electroencephalography (21, 22).

The objective of the present study was to determine the relationships between spatio-temporal gait parameters recorded during single task walking (STW) as well as dual task walking (DTW) and acute/chronic pain intensity, psychosocial aspects, executive functions as well as PFC activity during walking in CLBP patients.

We hypothesized that, due to the effect of CLBP on physical and cognitive functions, higher acute and chronic pain intensity is associated with shorter stride length, slower gait velocity and higher variability of these parameters. Given that the minimum toe clearance (MTC) and its variability do not seem to be affected by CLBP (14), no correlations with acute and chronic pain intensity were expected (i). Similar results were presumed for the associations between the mentioned spatio-temporal gait parameters and depression, kinesiophobia scores as well as the pain coping ability (ii). Additionally, since walking requires attention and EFs, the executive performance was expected to be positively correlated with stride length as well as gait velocity and negatively with their variability, but not with the MTC and its variability (iii). Lastly, based on the relationship between gait and attention as well as EFs, the PFC activity was assumed to correlate with stride length, gait velocity and their respective variabilities (iv).

## 2. Materials and methods

### 2.1. Participants and study design

The cross-sectional data presented in this article were acquired in the context of a longitudinal study investigating the effects of a multimodal exercise intervention on physical and cognitive functions in patients with CLBP (see study protocol for further information (23)). The presented data were recorded during the baseline measurements (German Clinical Trial Register, ID: DRKS00021696/10.07.2020^1^). The study has been approved by the ethics committee of the Medical Faculty of the Otto von Guericke University Magdeburg (OvGU; Germany) (No.: 182/18) and was conducted at the OvGU in cooperation with the Orthopaedic Department II of the Medical Care Centre Klinikum Magdeburg (Germany).

#### 2.1.1. Sample size

The sample size for the above-mentioned longitudinal study has been calculated using G*Power (version 3.1.9.7.). For the respective study, two groups and two covariates were planned. The calculation considered an α-level of 0.05 and a power of 0.95 resulting in a total sample size of 84 participants. Assuming a dropout rate of 15%, a sample size of 100 patients was considered appropriate (23).

#### 2.1.2. Recruitment process

The patients were recruited from July 2020 to January 2021 *via* an advertisement published in a local newspaper. In total, 243 CLBP patients were invited per telephone to the Medical Care Centre Klinikum Magdeburg for a medical anamnesis. There, the patients were treated by an orthopaedist and were recruited based on the following inclusion criteria: (i) ≥ 50 years old, (ii) average LBP score greater than 4 on a numeric rating scale (NRS; from 0 to 10 during the last 4 weeks), (iii) lasting LBP symptoms for more than 3 months and (iv) diagnosed to suffer from CLBP according to the International Classification of Diseases 10th revision (ICD-10: M54 Dorsalgia; M48.0 Spinal stenosis; M54.5 Low back pain; M54.4 Lumbago with sciatica; M54.1 Radiculopathy; M41.5 Other secondary scoliosis; M43.1 Spondylolisthesis; M42.1 Adult osteochondrosis of spine; M51.2 Other specified intervertebral disk displacement; M47.8 Other spondylosis; M53.2 Spinal instabilities).

CLBP patients were excluded if they: (i) had more than two previous spine surgeries, (ii) had more than three spinal segments fused, (iii) had any surgical intervention during the previous 6 months, (iv) were unable to walk without an aid, (v) had strength reductions higher than 25% according to Janda’s muscle function diagnostic (24), (vi) had any congenital spine deformities and (vii) had any neurological, cardiovascular, psychological and musculoskeletal diseases as well as vestibular disorders or dizziness that could impede the execution of the measurements.

Finally, 111 CLBP patients (81 females, 30 males) met the specific requirements. They were asked to sign a written informed consent and also received four questionnaires: The German version of the Tampa Scale of Kinesiophobia (TSK), the German Freiburger Questionnaire on Physical Activity, the German Pain Questionnaire (Deutsche Schmerzfragebogen, GPQ) and the Coping Strategies Questionnaire German version (CSQ). The questionnaires were used to assess the fear of movement related to pain, the level of daily activity, demographic and chronic pain related data (25) and finally to evaluate the effectiveness of coping strategies over pain (“control over pain” and “ability to decrease pain”) (26).

### 2.2. Experimental procedure

Approximately 3 weeks after the anamnesis, the patients were invited to the OvGU laboratory for the experimental session. They were asked to fill the above-mentioned questionnaires the day before. Because of the time delay, all criteria were again checked before the measurements. Among the 111 CLBP patients initially recruited, 108 (79 females, 29 males) participated in our experiment. Two patients had to be excluded due to their rating of chronic pain below 4 on the NRS and one due to dementia.

Firstly, the participants were informed in detail about the test protocol. Then, they completed four additional German questionnaires (i.e., the Oswestry Disability Index, the EuroQol Group EQ-5D, the Beck Depression Inventory II (BDI-II) and the Fatigue Subscale Profile of Mood States to assess the back pain associated disability in daily life, the health-related quality of life, the level of depression and state fatigue, respectively). Thereafter, the first executive performance test, the paper and pencil version of the Color-Word-Interference Test (27), was performed to assess the ability to inhibit cognitive interference (28). To reduce the cognitive demand at the beginning of the experiment, the second executive function test [i.e., Trail Making Test (TMT)] was conducted at the end of the session. Afterwards, the range of motion of the trunk was assessed using the mobee® med (SportMed A.G. SA, Luxembourg). Subsequently, the patients were equipped with a three-channel electrocardiogram (SOMNOtouch™ RESP, SOMNOmedics GmbH, Germany) and two inertial measurement units (IMU; Xsens Technologies B.V., Netherlands) required for the fNIRS measurements and gait performance assessment. Then, the patients were asked to complete two Timed Up-and-Go Test trials and one five-repetition sit-to-stand test trial on a force plate (Type 9260AA, Kistler Group, Winterthur, Switzerland).

Afterwards, the subjects were equipped with the fNIRS cap to assess the activity of the PFC’s subareas [i.e., dorsolateral prefrontal cortex (DLPFC), the frontopolar cortex (FPC) and the Broca]. These areas were, respectively, selected for their involvement in pain processing and executive functioning (18), attention and/or resource reallocation during concurrent tasks (29) and lastly, speech and executive functioning (30). Then, they performed the motor-cognitive tasks in a randomized order: STW and DTW (walking + arithmetic task). These trials were followed by additional randomly assigned static postural control tasks (i.e., standing with open and closed eyes as well as standing combined with an arithmetic task) on a force plate. Before and after performing all four tasks, the patients were asked to evaluate their current level of perceived fatigue on an NRS (0–10, from no fatigue to worst possible fatigue). At the end of this procedure, the fNIRS cap was removed.

Afterwards, the participants performed the TMT Part A and B as a measure of cognitive flexibility (31). The testing session ended with the six-minute walk test.

### 2.3. Equipment and outcome measures

To simplify the readability, only those parameters considered to be correlated with the spatio-temporal gait parameters are presented and discussed below. That means, the focus is on the pain related data, TSK, CSQ, BDI-II, EFs and the fNIRS data. For the questionnaires, the Freiburger Questionnaire on Physical Activity, the Oswestry disability index, the EuroQol and the Fatigue Subscale Profile of Mood States have been omitted. Additionally, the static postural control data as well as the physical tests (i.e., trunk mobility, Timed Up-and-Go Test, five-repetition sit-to-stand test, six-minute walk test) have also been omitted from this study.

#### 2.3.1. Acute/chronic pain, pain coping strategies and psychosocial factors

The intensity of acute and chronic pain was assessed with an NRS from 0 to 10 provided in the GPQ. To assess the fear of movement, we used the short version of the TSK (32) consisting of 11 items that should be scored on a 4-point Likert Scale (1 = “strongly disagree,” 2 = “somewhat disagree,” 3 = “somewhat agree,” 4 = “strongly agree”). The final score ranges from 11 to 44. For the coping strategies, the two subscales of the effectiveness ratings were considered (i.e., “control over pain” and “ability to decrease pain”). The latter were rated using an analog Likert Scale ranging from 0 to 6. Lastly, the depression level was evaluated using the BDI-II, a 21-item questionnaire with a final score ranging from 0 to 63. The depression level is categorized as follows: 0–13 minimal, 14–19 mild, 20–28 moderate and 29–63 severe depression (33).

#### 2.3.2. Executive functions

The EFs are defined as higher-order cognitive skills coordinating the subjects’ thoughts and actions towards the accomplishment of a specific goal (34) and they are usually separated into three categories: (i) inhibitory control, (ii) working memory and (iii) cognitive flexibility (35). To assess them, two different cognitive tasks have been conducted. Firstly, the paper version of the Color-Word-Interference Test by Bäumler (27) was performed to evaluate the ability to inhibit cognitive interference by calculating the interference score (IS) according to Stroop (36):


IS=T+2t×E

with T as the total time, t as the mean time per words (216 words) and E as the number of uncorrected errors. Therefore, a higher IS is associated with poorer inhibitory control. Secondly, cognitive flexibility has been assessed using the paper version of the TMT. The final score was quantified as the time difference between the parts B and A (37). Thus, a higher score is equivalent to poorer cognitive flexibility.

#### 2.3.3. Single task walking, dual task walking and arithmetic task

A block design was used for the fNIRS recordings (38). Following the recommendations by Herold et al. (39), the blocks consisted of 33 s of rest in a standing position (baseline) followed by the respective task performed over 30 s (activity). This was repeated four times starting and ending with a baseline (total time = 4:45 min). During STW and DTW, the patients were asked to walk back and forth on a 15 m track marked every 3 m. Walking (activity) and standing (baseline) were announced loudly by the test instructor. For the arithmetic task, a random number between 300 and 400 was given and the participants were asked to perform serial subtractions by 3. The total number of correct answers (i.e., the total number of answers minus the number of mistakes) was evaluated.

#### 2.3.4. Gait data recording and processing

The IMUs were placed on the proximal part of each foot. Based on 3D acceleration and gyroscope data, spatio-temporal gait parameters were calculated using the algorithm developed by Hamacher et al. (40). The outcome parameters of interest were stride length, gait velocity, MTC and their respective relative variability (coefficient of variation, CoV = 100 × standard deviation/mean). All data were processed in MATLAB (MathWorks®, Version R2020b, Natick, United States).

#### 2.3.5. fNIRS data recording and processing

The fNIRS system uses the properties of the oxygenated (HbO) and deoxygenated (HbR) hemoglobin to absorb light at different spectra, to measure relative changes in HbO and HbR concentrations in neuronal tissue, which is related to brain activity (39). To do so, the haemodynamic response in the PFC was recorded during walking using two sets of a continuous wave fNIRS systems (NIRSport, NIRx Medical Technologies, NY, United States), each connected to a cap of a different size (56 or 58 cm of circumference) (EasyCap GmbH, Herrsching, Germany). Head circumference was measured using a flexible measuring tape from the most prominent part of the forehead to the widest part of the back of the head. The smaller cap was used for head circumferences < 56.5 cm and the larger for ≥ 56.5 cm. Both sets were equipped with 8 sources, 8 detectors and 8 short separation channels with an average source-detector distance of 30 to 40 mm. The fNIRS system inherent wavelengths are 760 nm and 850 nm and the sampling rate is fixed at 7.81 Hz. The placement of each optode on the PFC followed the fNIRS optodes’ Location Decider toolbox (41). The sensitivity of the channels is documented in Broscheid et al. (42).

To assure an optimal fitting of the cap, the Cz point (according to the international 10–20 system for electroencephalograms) was centrally positioned between nasion to inion and between the left and right preauricular points. Additionally, a darkening cap was applied on top of the diodes to avoid interference from ambient light. This set up allowed to capture the haemodynamic signal of the, respectively, left, right and medial area of the DLPFC Brodmann area 9 and 46 (BA9, BA46) and FPC Brodmann area 10 (BA10) as well as the left and right Broca Brodmann area 45 (BA45). These subareas were composed of the following channels: left DLPFC BA9 (channels 17, 20 and 22), right DLPFC BA9 (channels 1, 18 and 21), medial DLPFC BA9 (channel 19), left DLPFC BA46 (channel 13), right DLPFC BA46 (channel 6), left FPC BA10 (channels 10, 11, 12 and 14), right FPC BA10 (channels 4, 5, 7 and 8), medial FPC BA10 (channel 9), left Broca BA45 (channels 15 and 16) and right Broca BA45 (channels 2 and 3).

All fNIRS data were processed using the open-source fNIRS software analysis package HomER3 (version 1.32.4) (43) and MATLAB (MathWorks®, Version R2020b, Natick, United States). We firstly used the *hmrR_PreprocessIntensity_NAN* function to suppress non-existing values, then the *hmrR_PruneChannels* function to exclude channels whose signal was either below $1\times 10^{-2}$ or above $1\times 10^{7}$ and also when the standard deviation was too high using signal to noise threshold at 2 and a source detector separation ranging from 0.0 to 45.0 mm. Afterwards, the raw signal was converted to optical density data using the *hmR_Intensity2OD* function. To minimize motion artifacts, the signal was filtered using the *hmR_MotionCorrectSplineSG* function (*p* = 0.99; frame size: 15 s) based on a spline interpolation and the Savitzky-Golay filter (44). A 3rd order Butterworth bandpass filter was also applied to remove physiological artifacts (45). To do so, the *Bandpass_Filter_OpticalDensity* function was used to filter out Mayer waves (low pass filter: 0.09 Hz) and to minimize the proportion of oscillations associated with vascular endothelial function (high pass filter: 0.01 Hz) (46). Finally, the optical density data were converted to HbO and HbR concentrations using the *hmrR_OD2Conc* function based on the Beer–Lambert Law adapting the differential path length factor to the age of each patient (47). Lastly, the *hmrR_GLM* function, a consecutive sequence of gaussian functions (width of the gaussian 0.5 and temporal spacing between consecutive gaussians 0.5), was applied to determine individual haemodynamic responses by the general linear model approach (48). For this function, the time range was set from −15 to 50 s and a 3rd order polynomial drift correction was applied. Each regression has been performed with the nearest short separation channel.

Afterwards, the data were post processed in MATLAB. The first 5 s have been cut out due to the time delay of the hemodynamic responses (49–51) and the last 5 s due to the expected ending (52). The remaining data (5–25 s of each interval) of each patient have been averaged and the channels were merged to the previously described subareas.

### 2.4. Statistical analysis

The statistical analysis has been performed using SPSS (IBM® SPSS® Statistics Version 27). Normal distribution of data was checked using the Kolmogorov-Smirnov test. Afterwards, correlation coefficients between the spatio-temporal gait parameters recorded during STW as well as DTW and the demographic data (age, height, weight), the questionnaire results (acute/chronic pain intensity, coping ability, kinesiophobia and depression level), the arithmetic performance, the executive function test performance (Color-Word-Interference Test, TMT) and the fNIRS data for each task have been calculated. When both parameters were normally distributed, the Pearson’s product moment correlation coefficient *r* was used. In all other cases, the Spearman’s rank-order correlation coefficient *ρ* was determined. Except for the fNIRS data, the MTC and its variability, all correlations have been calculated using one-tailed tests due to our directional hypotheses. Since multiple patterns of PFC hemodynamic have been observed during the execution of cognitive and/or motor tasks (12), a two-tailed test has been used for the correlation analysis between PFC activity and the spatio-temporal gait parameters. Additionally, the correlation coefficients have been interpreted according to Cohen (53) (i.e., *r* < 0.1: very small; 0.1 ≤ *r* < 0.29: small; 0.3 ≤ *r* < 0.5: moderate; r ≥ 0.5: large) with a significance level set at *p* < 0.05. Lastly, according to the results of the Kolmogorov-Smirnov test, either a paired *t*-test or a Wilcoxon rank test has been performed for the spatio-temporal gait parameters, the arithmetic task and the fNIRS data to assess the difference between the STW and DTW (see Supplementary Table S1).

## 3. Results

For every parameter presented below, the number of analyzed cases was given since some data were missing. This was either due to misreporting, unreadable data or poor-quality of raw data. The characteristics of the patients including the results of the questionnaires, the cognitive test performance and spatio-temporal gait parameters as well as the PFC activity during walking are, respectively, presented in Tables 1–3.

**Table 1:** Participants’ characteristics and questionnaires’ results.

	Means ± Standard Deviations
*Participants (n = 108)*	
Age (years)	67.7 ± 8.4
Weight (kg)	76.6 ± 16.2
Height (m)	1.67 ± 0.08
*Coping strategies Questionnaire (n = 104)*	
Ability to Decrease Pain	2.8 ± 0.8
Control Over Pain	3.1 ± 1.0
*Tampa Scale of Kinesiophobia (n = 105)*	
Total Score	22.2 ± 6.6
*German Pain Questionnaire (n = 108)*	
Acute Pain	4.4 ± 1.5
Chronic Pain	5.3 ± 1.2
*Beck Depression Inventory-II (n = 108)*	
Total Score	9.1 ± 5.9

**Table 2:** Descriptive data of cognitive test performance and spatio-temporal gait parameters.

	Means ± Standard Deviations
*Cognitive tests*	
IS (*n* = 105)	269.2 ± 77.8
TMT A [s] (*n* = 108)	38.2 ± 13.1
TMT B [s] (*n* = 108)	96.2 ± 43.4
TMT B-A [s] (*n* = 108)	58.0 ± 38.2
Arithmetic task (*n* = 103)	43.0 ± 14.4
Arithmetic task (DTW) (*n* = 105)	37.5 ± 13.7
*STW*	
Stride length [cm] (*n* = 93)	126.4 ± 12.7
Velocity [m/s] (*n* = 93)	1.2 ± 0.2
MTC [cm] (*n* = 96)	2.3 ± 0.7
Stride length CoV [%] (*n* = 93)	9.7 ± 2.2
Velocity CoV [%] (*n* = 93)	11.7 ± 2.2
MTC CoV [%] (*n* = 96)	27.5 ± 9.9
*DTW*	
Stride length [m] (*n* = 91)	121.8 ± 13.8
Velocity [m/s] (*n* = 91)	1.1 ± 0.2
MTC [cm] (*n* = 94)	2.0 ± 0.6
Stride length CoV [%] (*n* = 91)	10.1 ± 2.6
Velocity CoV [%] (*n* = 91)	12.6 ± 2.9
MTC CoV [%] (*n* = 94)	28.4 ± 9.7

IS: Interference score; TMT: Trail Making Test; STW: Single task walking; DTW: Dual task walking; MTC: Minimum toe clearance; CoV: Coefficient of variation.

**Table 3:** Descriptive data of the prefrontal cortex haemodynamics.

	HbO [μmol/L]	HbR [μmol/L]
*STW (n = 88)*		
rDLPFC (BA9)	0.54 ± 1.61	-0.02 ± 0.42
rDLPFC (BA46)	0.99 ± 2.19	-0.37 ± 0.60
lDLPFC (BA9)	0.60 ± 1.66	0.05 ± 0.39
lDLPFC (BA46)	1.15 ± 1.94	-0.24 ± 0.61
rFPC (BA10)	0.87 ± 1.94	-0.18 ± 0.48
lFPC (BA10)	0.70 ± 1.78	-0.18 ± 0.48
rBroca (BA45)	1.07 ± 2.14	-0.16 ± 0.54
lBroca (BA45)	1.09 ± 1.93	-0.14 ± 0.68
mFPC (BA10)	0.18 ± 1.78	-0.17 ± 0.46
mDLPFC (BA9)	0.37 ± 2.14	-0.11 ± 0.50
*DTW (n = 88)*		
rDLPFC (BA9)	1.80 ± 2.21	-0.06 ± 0.53
rDLPFC (BA46)	1.83 ± 2.56	-0.51 ± 0.87
lDLPFC (BA9)	1.83 ±2.07	0.03 ± 0.55
lDLPFC (BA46)	1.92 ± 2.49	-0.35 ± 0.82
rFPC (BA10)	1.88 ± 2.14	-0.22 ± 0.72
lFPC (BA10)	1.90 ± 2.30	-0.19 ± 0.75
rBroca (BA45)	2.39 ± 2.48	-0.18 ± 0.74
lBroca (BA45)	2.25 ± 2.30	-0.22 ± 0.75
mFPC (BA10)	0.84 ± 2.31	-0.19 ± 0.59
mDLPFC (BA9)	1.43 ± 2.42	-0.17 ± 0.65

Values are presented as means ± standard deviations.

STW: Single task walking; DTW: Dual task walking; rDLPFC: Right dorsolateral prefrontal cortex; lDLPFC: Left dorsolateral prefrontal cortex; rFPC: Right frontopolar cortex; lFPC: Left frontopolar cortex; rBroca: Right Broca; lBroca: Left Broca; mFPC: Medial frontopolar cortex; mDLPFC: Medial dorsolateral prefrontal cortex; HbO: Oxygenated haemoglobin; HbR: Deoxygenated haemoglobin.

### 3.1. Correlations between spatio-temporal gait parameters and participants’ characteristics

All correlation coefficients between the spatio-temporal gait parameters and the participants’ characteristics are shown in Table 4. For the STW condition, age showed a small negative correlation with stride length and a positive small correlation with the velocity’s variability resulting in older patients having shorter stride length and higher gait variability. Height displayed a small positive correlation with the stride length, MTC, stride length CoV and velocity CoV indicating that taller patients had longer stride length, a higher MTC and a greater gait variability. Weight also showed a small negative correlation with velocity resulting in heavier patients walking slower. For the DTW condition, the associations of spatio-temporal gait parameters with age were analogous. Height was positively correlated with all three spatio-temporal gait parameters but not with their respective variability. Thus, taller patients displayed longer stride length, faster velocity and a higher MTC during DTW. Lastly, weight demonstrated a moderate positive correlation with the MTC meaning that heavier patients had a higher MTC.

**Table 4:** Relationships between gait parameters and the participants’ characteristics.

Parameters	Age			Height			Weight		
	*n*	r/ρ	*p*	*n*	r/ρ	*p*	*n*	r/ρ	*p*
*STW*									
Stride length	97	**−0.230#**	**0.012**	97	**0.237§**	**0.001**	93	−0.028#	0.395
Velocity	97	−0.157#	0.062	97	−0.002§	0.490	93	**−0.195#**	**0.031**
MTC	97	−0.137§	0.182	97	**0.262§**	**0.009**	93	0.145§	0.165
Stride length CoV	97	0.162#	0.057	97	**0.199$**	**0.025**	93	0.120#	0.126
Velocity CoV	97	**0.238#**	**0.009**	97	**0.218§**	**0.016**	93	0.137#	0.095
MTC CoV	97	−0.131§	0.202	97	0.038§	0.714	93	−0.035§	0.742
*DTW*									
Stride length	91	**−0.202#**	**0.027**	91	**0.352§**	**0.000**	88	0.104#	0.167
Velocity	91	−0.140#	0.093	91	**0.217§**	**0.019**	88	0.061#	0.287
MTC	94	−0.039#	0.706	94	**0.369§**	**0.000**	91	**0.317#**	**0.002**
Stride length CoV	91	**0.202§**	**0.027**	91	0.172§	0.051	88	−0.038§	0.362
Velocity CoV	91	**0.266§**	**0.005**	91	0.131§	0.108	88	−0.034§	0.376
MTC CoV	93	−0.043§	0.649	93	0.076§	0.468	90	−0.086§	0.420

STW, Single task walking; DTW, Dual task walking; MTC, Minimum toe clearance; CoV, Coefficient of variation; §, Spearman’s rank-order correlation ρ; #, Pearson’s product moment correlation coefficient *r*, p, *p*-value; Bold: *p* < 0.05.

### 3.2. Correlations between spatio-temporal gait parameters and acute/chronic pain intensity, pain coping strategies as well as psychosocial factors

The chronic pain intensity, kinesiophobia and the “control over pain” skill did not show any significant correlation with the spatio-temporal gait parameters recorded during STW and DTW. Regarding the level of acute pain, only the variability of velocity during DTW demonstrated a small negative correlation. Therefore, patients suffering from stronger pain walked with less variable velocity. The “ability to reduce pain” was negatively associated with the variability of velocity during STW, while it was negatively correlated with stride length and velocity in the DTW condition. Consequently, patients with better ability to reduce pain demonstrated lesser variability, shorter stride length and slower velocity. Lastly, the depression level was only negatively correlated with the velocity during DTW with a higher depression level resulting in a slower gait velocity (Table 5).

**Table 5:** Relationships between gait parameters and acute/chronic pain, depression, kinesiophobia and the pain coping strategies.

	Acute pain			Chronic Pain			BDI-II			TSK			CSQ Ability to reduce pain			CSQ Control Over Pain		
Parameters	*n*	ρ	*p*	*n*	ρ	*p*	*n*	ρ	*p*	*n*	ρ	*p*	*n*	ρ	*p*	*n*	ρ	*p*
*STW*																		
Stride length	96	-0.002	.493	97	-0.085	.204	97	-0.102	.159	94	-0.062	.276	94	-0.068	.258	94	0.039	.356
Velocity	96	0.090	.191	97	0.004	.486	97	-0.070	.247	94	-0.088	.198	94	-0.032	.379	94	0.120	.124
MTC	96	-0.032	.755	97	0.084	.413	97	-0.108	.291	94	0.024	.817	94	-0.020	.850	94	-0.095	.362
Stride length CoV	96	0.052	.307	97	0.063	.271	97	-0.122	.117	94	-0.056	.295	94	-0.166	.055	94	0.030	.386
Velocity CoV	96	0.018	.432	97	0.004	.486	97	-0.135	.094	94	-0.052	.310	94	**-0.176**	**.045**	94	-0.017	.436
MTC CoV	96	-0.002	.985	97	-0.063	.541	97	0.091	.377	94	-0.007	.946	94	-0.057	.583	94	0.027	.798
*DTW*																		
Stride length	90	-0.118	.133	91	-0.111	.147	91	-0.097	.181	88	0.018	.436	88	**-0.210**	**.025**	88	-0.008	.472
Velocity	90	0.016	.441	91	-0.018	.434	91	**-0.195**	**.032**	88	0.031	.389	88	**-0.221**	**.019**	88	0.029	.394
MTC	92	-0.055	.605	94	0.010	.924	94	-0.106	.309	91	-0.014	.896	90	-0.145	.173	90	-0.094	.377
Stride length CoV	90	-0.148	.082	91	-0.017	.435	91	-0.118	.133	88	-0.006	.480	88	-0.063	.279	88	0.043	.346
Velocity CoV	90	**-0.189**	**.037**	91	-0.069	.258	91	-0.103	.167	88	-0.129	.115	88	-0.102	.171	88	0.012	.455
MTC CoV	92	-0.060	.575	93	-0.091	.387	94	0.044	.675	90	0.022	.839	90	-0.126	.239	89	-0.041	.703

TSK: Tampa Scale of Kinesiophobia; CSQ: Coping Strategies Questionnaire; STW: Single task walking; DTW: Dual task walking; MTC: Minimum toe clearance; CoV: Coefficient of variation; ρ: Spearman’s rank-order correlation *ρ*; *p*: *p*-value; Bold: *p* < 0.05.

### 3.3. Correlations between spatio-temporal gait parameters, executive functions and arithmetic task performance

**Figure 1:**
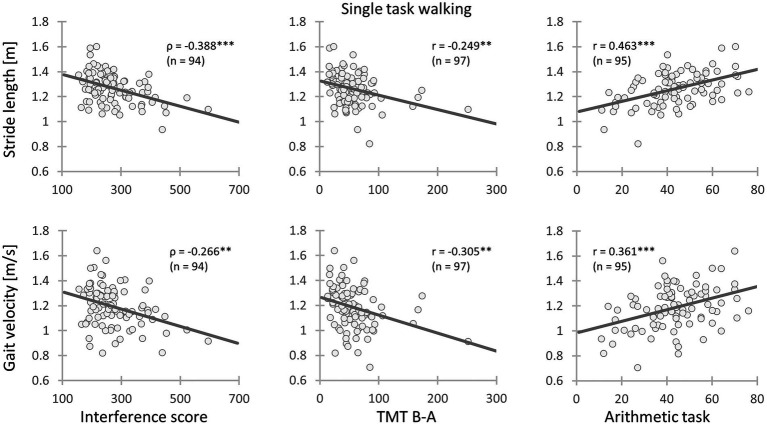
Relationships between gait parameters and executive functions as well as the arithmetic task performance. **Left**: Chronic low back pain (CLBP) patients with less inhibitory interference had longer stride length and faster gait velocity during single task walking; **Mid**: CLBP patients with better cognitive flexibility had longer stride length and faster gait velocity during single task walking; **Right**: CLBP patients with a higher arithmetic task performance had longer stride length and faster gait velocity during single task walking (IS, Interference score; TMT, Trail Making Test performance; ρ, Spearman’s rank-order correlation coefficient; *r*, Pearson’s product moment correlation coefficient; *: 0.01 ≤ *p* < 0.05; **: 0.001 ≤ *p* < 0.01; ***: *p* < 0.001).

In the STW and DTW conditions, both stride length and velocity were associated with the EFs. Similarly, the performance during the arithmetic task displayed analogous results. Therefore, patients with better cognitive performance demonstrated longer strides and faster gait velocity (see Table 6; Figure 1). Moreover, arithmetic task performance showed a small positive correlation with the stride length CoV during STW.

**Table 6:** Relationships between gait parameters and executive functions as well as the arithmetic task performance.

	IS			TMT B-A			Arithmetic task		
Parameters	*n*	r/ρ	*p*	*n*	r/ρ	*p*	*n*	r/ρ	*p*
*STW*									
Stride length	94	**−0.388§**	**<0.001**	97	**−0.249§**	**0.007**	95	**0.463#**	**<0.001**
Velocity	94	**−0.266§**	**0.005**	97	**−0.305§**	**0.001**	95	**0.361#**	**<0.001**
MTC	94	−0.034§	0.742	97	−0.015§	0.885	95	0.105§	0.309
Stride length CoV	94	0.035§	0.370	97	0.039§	0.351	95	**0.202#**	**0.025**
Velocity CoV	94	−0.088§	0.200	97	0.092§	0.186	95	0.150#	0.074
MTC CoV	94	−0.003§	0.974	97	−0.009§	0.931	95	0.080§	0.442
*DTW*									
Stride length	89	**−0.285§**	**0.003**	91	**−0.242$**	**0.010**	89	**0.425#**	**<0.001**
Velocity	89	**−0.323§**	**0.001**	91	**−0.260$**	**0.006**	89	**0.272#**	**0.005**
MTC	92	−0.043§	0.686	94	−0.080$	0.446	92	0.143#	0.173
Stride length CoV	89	0.043§	0.343	91	0.088§	0.204	89	0.085§	0.213
Velocity CoV	89	−0.039§	0.358	91	0.118§	0.132	89	0.025§	0.407
MTC CoV	92	−0.113§	0.288	94	0.045§	0.666	92	0.086§	0.417

STW, Single task walking; DTW, Dual task walking; IS, Interference score; TMT, Trail Making Test; MTC, Minimum toe clearance; CoV, Coefficient of variation. §, Spearman’s rank-order correlation ρ; #, Pearson’s product moment correlation coefficient *r*; p, *p*-value; Bold: *p* < 0.05.

### 3.4. Correlations between spatio-temporal gait parameters and PFC activity

**Figure 2:**
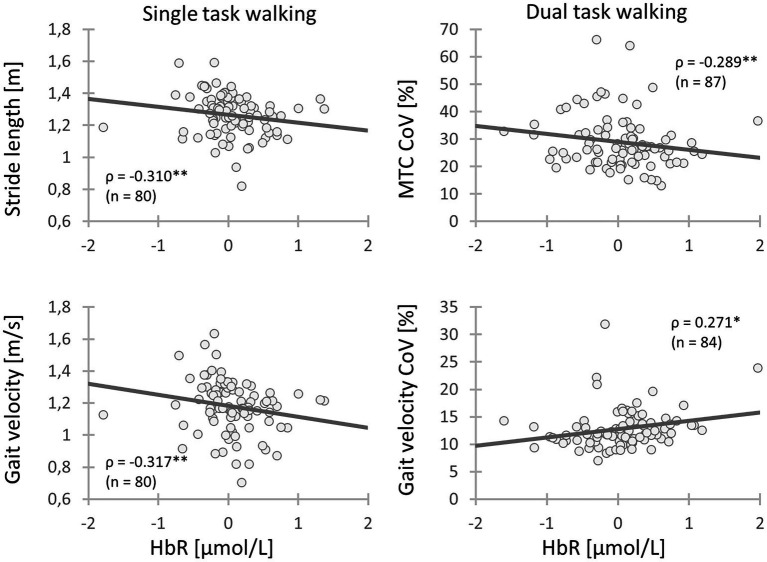
Relationships between gait parameters and activity of the left DLPFC (BA9) during walking. Chronic low back pain (CLBP) patients with a higher activity of the left DLPFC (BA9) during walking had longer stride length, faster gait velocity, less variability of gait velocity and higher MTC variability (CLBP, Chronic low back pain; MTC, Minimum toe clearance; CoV, Coefficient of variation; HbR, Deoxygenated hemoglobin; ρ, Spearman’s rank-order correlation ρ; *: 0.01 ≤ *p* < 0.05; **: 0.001 ≤ *p* < 0.01; ***: *p* < 0.001).

The HbO concentration did not show any association with the spatio-temporal gait parameters recorded during STW. However, the HbR concentration in the left DLPFC (BA9) displayed a moderate negative correlation with stride length (ρ = −0.310, *p* = 0.005) and velocity (ρ = −0.317, *p* = 0.004) indicating that higher left DLPFC (BA9) activity resulted in longer stride length and faster gait velocity. The HbR concentration of the left DLPFC (BA46) and left Broca (BA45) recorded during DTW showed a small positive correlation with gait velocity (ρ = 0.248, *p* = 0.030 and ρ = 0.239, *p* = 0.036, respectively). This suggests that an increment of left DLPFC (BA46) and left Broca (BA45) activity is associated with faster walking velocity during DTW. Regarding the HbR concentration during DTW, the right DLPFC (BA9) showed a small positive correlation with the MTC (*r* = 0.297, *p* = 0.007). Thus, the higher the activity of the right DLPFC (BA9), the lower was the MTC. Lastly, the left DLPFC (BA9) showed a small positive correlation with the velocity CoV (ρ = 0.271, *p* = 0.017) and a small negative correlation with the MTC CoV (ρ = −0.289, *p* = 0.010). This indicates that a higher left DLPFC (BA9) activity is associated with lesser velocity variability but greater MTC variability (Table 7 and Figure 2).

**Table 7:** Relationships between gait parameters and prefrontal cortex haemodynamic.

	rDLPFC (BA9)	rDLPFC (BA46)	lDLPFC (BA9)	lDLPFC (BA46)	rFPC (BA10)	lFPC (BA10)	rBroca (BA45)	lBroca (BA45)	mFPC (BA10)	mDLPFC (BA9)
STW										
*HbO*										
Stride Length (n = 80)	-0.025§	0.199#	-0.154§	-0.031§	-0.022§	-0.136§	0.016§	-0.157#	-0.058§	-0.038§
Velocity (n = 80)	-0.075§	0.108#	-0.122§	0.004§	-0.075§	-0.180§	-0.044§	-0.186#	-0.190§	-0.047§
MTC (n = 80)	-0.049§	0.080§	-0.069§	0.010§	-0.032§	-0.219§	-0.096§	-0.012§	-0.045§	-0.166§
Stride Length CoV (n = 80)	0.128§	0.016#	0.172§	0.104§	0.164§	0.128§	0.123§	-0.045#	0.164§	0.073§
Velocity CoV (n = 80)	0.120§	0.023#	0.150§	0.032§	0.132§	0.122§	0.113§	-0.066#	0.214§	0.089§
MTC CoV (n = 80)	-0.003§	-0.018§	-0.041§	-0.185§	0.038§	0.022§	0.037§	-0.056§	0.116§	0.095§
*HbR*										
Stride Length (n = 80)	-0.189#	0.096#	-0.310**§	-0.027#	0.011#	-0.153#	-0.149§	-0.121§	-0.002#	-0.181§
Velocity (n = 80)	-0.176#	0.090#	-0.317**§	0.073#	0.014#	-0.080#	-0.168§	-0.061§	0.035#	-0.199§
MTC (n = 80)	0.076§	0.007§	-0.081§	0.012§	0.028§	0.036§	0.096§	0.018§	0.026§	0.112§
Stride Length CoV (n = 80)	0.127#	-0.065#	0.060§	-0.122#	-0.037#	0.093#	-0.038§	0.064§	0.050#	0.100§
Velocity CoV (n = 80)	0.187#	0.042#	0.097§	-0.061#	0.032#	0.127#	-0.015§	0.027§	0.076#	0.105§
MTC CoV (n = 80)	-0.165§	0.072§	-0.077§	-0.015§	0.014§	-0.001§	-0.057§	-0.042§	-0.105§	-0.124§
DTW										
*HbO*										
Stride Length (n = 84)	0.069§	0.013§	0.134§	0.197§	0.197#	0.061§	0.070§	0.209§	0.076§	0.162§
Velocity (n = 84)	0.014§	-0.049§	0.094§	0.248*§	0.201#	0.122§	-0.035§	0.239*§	-0.048§	0.102§
MTC (n = 87)	0.141§	0.009§	0.110§	0.199§	-0.079#	0.092§	-0.060§	0.205§	-0.104§	0.085§
Stride Length CoV (n = 84)	0.094§	0.007§	0.052§	-0.024§	-0.064§	-0.159§	-0.020§	-0.105§	-0.116§	0.129§
Velocity CoV (n = 84)	0.092§	0.053§	0.086§	-0.010§	-0.137§	-0.139§	-0.046§	-0.097§	-0.036§	0.182§
MTC CoV (n = 87)	0.185§	0.179§	0.150§	-0.044§	0.059§	0.069§	0.144§	0.003§	0.038§	0.217§
*HbR*										
Stride Length (n = 84)	0.025#	0.024#	-0.069#	-0.032§	0.081§	0.110#	-0.004§	0.002#	-0.091#	-0.081§
Velocity (n = 84)	0.023#	-0.078#	-0.093#	0.060§	0.084§	0.097#	0.057§	-0.007#	-0.130#	-0.029§
MTC (n = 87)	0.297**#	-0.026#	0.155#	0.044§	0.145§	0.134#	0.061§	0.088#	0.088#	0.216§
Stride Length CoV (n = 84)	0.119§	0.035§	0.150§	0.009§	0.006§	0.139§	-0.044§	0.036§	0.099§	0.153§
Velocity CoV (n = 84)	0.190§	0.093§	0.271*§	0.132§	0.093§	0.214§	-0.001§	0.002§	0.157§	0.221§
MTC CoV (n = 87)	-0.209§	0.116§	-0.289**§	-0.082§	0.105§	-0.002§	0.046§	-0.144§	-0.083§	-0.154§

rDLPFC: Right dorsolateral prefrontal cortex, lDLPFC: Left dorsolateral prefrontal cortex; mDLPFC; Medial dorsolateral prefrontal cortex; rFPC: Right frontopolar cortex; lFPC: Left frontopolar cortex; mFPC: Medial frontopolar cortex; BA: Brodmann area; HbO: Oxygenated haemoglobin; HbR: Deoxygenated haemoglobin; STW: Single task walking; DTW: Dual task walking; MTC: Minimum toe clearance; CoV: Coefficient of variation; §: Spearman’s rank-order correlation *ρ*; #: Pearson’s product moment correlation coefficient *r;* *: .01 < *p* < .05; **: .001 < *p* < .01; Bold: p < 0.05.

## 4. Discussion

This cross-sectional study investigated the relationships between spatio-temporal gait parameters recorded during STW as well as DTW and the acute/chronic pain intensity, psychosocial aspects, executive functions and PFC activity while walking in CLBP patients. First, it was hypothesized that acute and chronic pain intensity would be associated with shorter stride length, slower gait velocity and higher gait variability. Second, it was assumed that higher depression and kinesiophobia as well as lower pain coping ability would be similarly associated with the spatio-temporal gait parameters. Third, higher executive performance was thought to be associated with longer stride length, faster gait velocity and lower variability. Finally, PFC activity was expected to be associated with the spatio-temporal gait parameters.

Data analysis did not reveal any associations between chronic pain and spatio-temporal gait parameters. Only acute pain was slightly negatively correlated with gait velocity CoV during DTW, which was not expected. Second, contrary to our hypothesis, it was found that the better the “ability to reduce pain” (CSQ), the lower the gait velocity CoV during STW and the shorter the stride length as well as the slower the gait velocity during DTW. For kinesiophobia, no correlations with spatio-temporal gait parameters were observed. However, consistent with our hypothesis, higher depression was associated with slower gait velocity during DTW. Third, as initially supposed, the better the CLBP patients performed in the executive function tests (IS, TMT) and the arithmetic task, the better was the gait performance (i.e., greater stride length and faster gait velocity) during STW and DTW. Furthermore, mainly the activity of the DLPFC was correlated with the spatio-temporal gait parameters. More precisely, a higher activity of the left DLPFC (BA9) during STW and the left DLPFC (BA46) as well as left Broca (BA45) during DTW were associated with higher gait velocity. The activity of the left DLPFC (BA9) during STW was further related to longer stride length. Moreover, it was found that the lower the activity of the right DLPFC (BA9), the higher the MTC during DTW. Finally, it was observed that the lower the activity in the left DLPFC (BA9), the higher the gait velocity CoV and the lower the MTC CoV during DTW.

### 4.1. Correlations between spatio-temporal gait parameters and acute/chronic pain intensity as well as psychosocial factors

In this study, chronic pain intensity was not associated with the spatio-temporal gait parameters and their variability during STW and DTW in CLBP patients. This result is not surprising, as the review by Koch and Hänsel (8) showed controversial results regarding the comparison of gait performance between CLBP patients and healthy controls during overground walking. Nevertheless, there is evidence that gait variability is higher in CLBP patients compared to healthy controls (8) indicating that chronic pain intensity might be related to a higher gait variability, which is not supported by our data. Of note, these studies varied in the examined gait parameters, the measurement devices (IMUs, motion capturing, instrumented walkways, etc.), the distance walked and whether chronic and/or acute pain intensity was recorded. Moreover, these studies performed only STW and compared gait performance between people with and without CLBP, but did not correlate pain intensity with gait measures of CLBP patients.

Regarding acute pain, a negative correlation was found with velocity CoV during DTW. This relationship might be interpreted as an avoidance or minimization of pain by consciously adapting the gait pattern to reduce unwanted movements (54, 55). Importantly, the influence of pain on gait measures also depends on the measurement environment. For instance, it was demonstrated that trunk variability and acute pain intensity in CLBP patients only correlated, when recorded in an environment of daily living but not on a treadmill in the laboratory (56).

Kinesiophobia and depression showed none to small associations with gait performance, respectively. Although, these psychosocial factors are thought to contribute to the development of CLBP (57), they were not or only slightly related to the spatio-temporal gait parameters in our sample. This is in accordance with the outcome of a recently published systematic review with meta-analysis, which found only very small to small pooled correlation coefficients between psychological factors (e.g., pain-related fear, catastrophizing, depression, anxiety and self-efficacy) and spinal movement amplitude as well as trunk muscle activity (58).

Interestingly, the “ability to reduce pain” was negatively correlated with stride length CoV during STW as well as stride length and velocity during DTW. These data might indicate that people with a better ability to reduce pain adopt a gait pattern with smaller stride length variability, shorter stride length and slower gait velocity to minimize pain associated with walking.

### 4.2. Correlations between spatio-temporal gait parameters, executive functions and the arithmetic task performance

Yogev-Seligmann et al. (11) already highlighted the relationships between cognitive functions and gait performance. Our results corroborate these findings and indicate that cognitive performance (i.e., inhibitory control, cognitive flexibility and arithmetic task performance) was slightly to moderately correlated with gait performance (i.e., longer stride length and faster gait velocity) in CLBP patients. Since arithmetic task performance (i.e., subtractions) partly depends on working memory (59, 60), it might be supposed that all three core EFs (i.e., inhibitory control, working memory and cognitive flexibility (35)) are required for optimal gait performance in CLBP. This is in agreement with the notion that CLBP patients exhibit a stronger cognitive regulation of gait coordination compared to healthy controls indicating a decreased automaticity of gait control (16).

### 4.3. Correlations between spatio-temporal gait parameters and PFC activity

Although brain activity was measured in several brain areas of the PFC, the most relevant brain regions are the DLPFC (BA9 and BA46), because of its involvement in pain processing and executive functioning (18), the FPC (BA10), since it is required for goal monitoring and control of cognitive resources (61) and lastly the Broca (BA45), due to its involvement in speech and executive functioning (30). To simplify the following discussion, the distinction between HbO and HbR will be ignored and instead the hemodynamic changes will be directly interpreted as changes in cortical activity.

Data analysis revealed only significant small to moderate correlations of the gait parameters with the activity in the DLPFC (BA9 and BA46) and left Broca (BA45) during STW and DTW in CLBP patients. Accordingly, an increased brain activity in the left DLPFC (BA9) was moderately related to longer stride length and a faster gait velocity during STW. This is in contrast to the results of Holtzer et al. (62) who have not found associations between brain activity in the PFC and gait velocity as well as stride length during STW in healthy older adults. This discrepancy is probably due to structural and functional changes associated with CLBP (63), which might require higher compensatory brain activity for successful gait performance even during STW. Given that the DLPFC is involved in cognitive, affective and sensory processing and shows an abnormally increased activity in chronic pain populations (64), the association between left DLPFC activity and gait measures might additionally reflect the processing of painful stimuli during STW.

During DTW, an increased activity in the left DLPFC (BA46) and left Broca (BA45) was associated with higher gait velocity. Although Holtzer et al. (62) have not found a relationship between HbO in the PFC and gait velocity in older adults, they revealed a positive relationship between PFC activity and stride length recorded during DTW. Similarly, Clark et al. (65) have found that older subjects with a larger increase in PFC activity performed better during complex walking tasks (e.g., walking over obstacles). Thus, the higher activity in the left DLPFC might reflect a better cognitive functioning and/or pain processing (64) during DTW in CLBP patients, which probably contributed to the better gait performance. The positive association between activity of the left Broca (BA45) and gait velocity might not only reflect speech during DTW but also a better executive function capacity (30) potentially contributing to the better gait performance.

These findings collectively indicate that the availability and utilization of prefrontal brain resources are crucial for optimal STW (i.e., stride length, gait velocity) and DTW (i.e., gait velocity) performance in CLBP patients. This assumption is further corroborated by the finding that a lower activity in the left DLPFC (BA9) was associated with a higher velocity CoV in the DTW condition indicating that a reduced brain activity capacity promotes a poorer gait performance.

In contrast, a lower activity of the right DLPFC (BA46) was associated with higher MTC values during DTW indicating a better gait performance, since lower MTC values are assumed to increase the risk of tripping (66). Given the fact that other gait parameters but not the MTC worsened in older adults when adding a cognitive task to overground walking (40), it can be speculated that the control of the MTC is highly automatized and hardly requires prefrontal resources. Therefore, it could be speculated that a higher degree of automaticity, indicated by lower brain activity is related to larger MTC values in CLBP patients. This assumption is further corroborated by the finding that a higher activity in the left DLPFC (BA9) was associated with a greater MTC CoV in the DTW condition meaning that an increased prefrontal contribution and less automaticity is associated with higher variability of the MTC, which can increase the risk of tripping.

These findings collectively indicate that the associations between PFC activity and gait measures probably depend on the level of automaticity. While gait measures susceptible to dual task interference, such as gait velocity, were positively related to brain activity during DTW in CLBP patients, this association was reversed for the MTC and its variability.

## 5. Limitations

Although correlations between gait performance and several aspects were found, these do not necessarily indicate causal relationships. Moreover, our patients had a higher age and, therefore, the results might not be valid for younger CLBP patients.

## 6. Conclusion

A good walking performance is crucial for safe locomotion and activities of daily living. The present study investigated the associations between gait performance and pain intensity, psychosocial aspects, executive functions and the PFC activity while walking in CLBP patients. The understanding of the multiple factors, which could impair gait performance, can help clinicians and therapists in the treatment and rehabilitation of CLBP patients. Although the found associations do not indicate causality, it seems that interventions that reduce depression and increase executive functioning might be suitable to improve gait performance in CLBP patients.

## Data availability statement

The raw data supporting the conclusions of this article will be made available by the authors, without undue reservation.

## Ethics statement

The studies involving human participants were reviewed and approved by Medical Faculty of the OvGU Magdeburg. The patients/participants provided their written informed consent to participate in this study.

## Author contributions

TN: investigation, documentation, data analysis, original draft preparation and review and editing. MB: conceptualization, methodology, documentation, data analysis, original draft preparation and review and editing. K-CB: conceptualization, methodology, investigation, documentation, data analysis, original draft preparation and review and editing. RB: conceptualization, methodology, investigation, documentation and review and editing. SW: investigation, documentation, data analysis and review and editing. SL, VM, LB, XM, and LJ: investigation, documentation and review and editing. AW, KM, and SD: patient recruitment, documentation and review and editing. JF and LS: funding acquisition, conceptualization, methodology, data analysis and review and editing. All authors contributed to the article and approved the submitted version.

## Funding

MultiMove Project (number ZS/2019/10/101921) funded by the European Regional Development Fund (project code: ajdx9exId4 EFRE 11.000sz00.00.0. 19 133780 0).

## Conflict of interest

The authors declare that the research was conducted in the absence of any commercial or financial relationships that could be construed as a potential conflict of interest.

## Publisher’s note

All claims expressed in this article are solely those of the authors and do not necessarily represent those of their affiliated organizations, or those of the publisher, the editors and the reviewers. Any product that may be evaluated in this article, or claim that may be made by its manufacturer, is not guaranteed or endorsed by the publisher.

## Footnotes

^1^ https://www.drks.de/drks_web/navigate.do?navigationld=trial.HTML&TRIAL_ID=DRKS00021696
