# Supplementary material for: Associations between gait performance and pain intensity, psychosocial factors, executive functions as well as prefrontal cortex activity in chronic low back pain patients: A cross-sectional fNIRS study
Source: Front Med (Lausanne). 2023 May 5;10:1147907. doi: 10.3389/fmed.2023.1147907 (PMC10196398; doi:10.3389/fmed.2023.1147907)
Supplement: Supplementary file 1 [file Table_1.docx]

*Supplementary Table*

Comparison of arithmetic task performance, gait parameters, and brain activity between single and dual tasks walking

|  |  | *n* | *t* | Z | *p* |
| --- | --- | --- | --- | --- | --- |
| Arithmetic task# | | 102 | 7.156 |  | < .000 |
| *Spatio-temportal gait parameters* | | | | | |
|  | Stride length [cm]# | 90 | 6.914 |  | < .000 |
|  | Velocity [m/s] | 90 | 10.220 |  | < .000 |
|  | MTC [cm] | 93 |  | -6.079 | < .000 |
|  | Stride length CoV | 90 |  | -0.268 | .789 |
|  | Velocity CoV | 90 |  | -2.440 | .015 |
|  | MTC CoV | 93 |  | -1.588 | .112 |
| *HbO* | | | | | |
|  | rDLPFC (BA9) | 94 |  | -4.719 | < .000 |
|  | rDLPFC (BA46) | 94 |  | -2.642 | .008 |
|  | lDLPFC (BA9) | 94 |  | -4.553 | < .000 |
|  | lDLPFC (BA46) | 94 |  | -2.219 | .026 |
|  | rFPC (BA10) | 94 |  | -4.244 | < .000 |
|  | lFPC (BA10) | 94 |  | -4.029 | < .000 |
|  | rBroca (BA45) | 94 |  | -4.180 | < .000 |
|  | lBroca (BA45) | 94 |  | -3.890 | < .000 |
|  | mFPC (BA10) | 94 |  | -1.989 | .047 |
|  | mDLPFC (BA9) | 94 |  | -2.947 | .003 |
| *HbR* | | | | | |
|  | rDLPFC (BA9)# | 94 | 0.503 |  | .616 |
|  | rDLPFC (BA46)# | 94 | 1.698 |  | .093 |
|  | lDLPFC (BA9) | 94 |  | -0.040 | .968 |
|  | lDLPFC (BA46) | 94 |  | -1.823 | .068 |
|  | rFPC (BA10) | 94 |  | -0.058 | .953 |
|  | lFPC (BA10)# | 94 | 0.106 | -0.126 | .916 |
|  | rBroca (BA45) | 94 |  | -0.255 | .799 |
|  | lBroca (BA45) | 94 |  | -1.480 | .139 |
|  | mFPC (BA10)# | 94 | 0.062 |  | .951 |
|  | mDLPFC (BA9) | 94 |  | -1.167 | .243 |
| STW: Single task walking; DTW: Dual task walking; MTC: Minimum toe clearance; #: Normally distributed; *t*: Paired *t*-test; Z: z-score; *p*: *p*-value | | | | | |
